# Anticancer Potential of *Lacticaseibacillus rhamnosus* in Colorectal Cancer—A Systematic Review of In Vitro Cell Culture Evidence

**DOI:** 10.3390/ijms27072944

**Published:** 2026-03-24

**Authors:** Arshiya Nasreen Bint Shajahan, Sakina Mustafa Vakhariya, Malak Moones Abedi, Syeda Nishaat Fatima, Liyan Khadeeja, Elham Hassan Nazari Fard, Abshina Shajahan, Vijaya Paul Samuel, Grisilda Vidya Bernhardt, Suresh Kumar Srinivasamurthy

**Affiliations:** 1 RAK College of Medical Sciences, RAK Medical and Health Sciences University, Ras Al Khaimah P.O. Box 11172, United Arab Emirates; arshiya.21901039@rakmhsu.ac.ae (A.N.B.S.); sakina.21901001@rakmhsu.ac.ae (S.M.V.); malaek.21901100@rakmhsu.ac.ae (M.M.A.); syeda.21901032@rakmhsu.ac.ae (S.N.F.); liyan.khadeeja22@gmail.com (L.K.); elham1601@icloud.com (E.H.N.F.); 2 College of Medicine, University of Sharjah, Sharjah P.O. Box 27272, United Arab Emirates; abshinashajahan@gmail.com; 3 Department of Anatomy, RAK College of Medical Sciences, RAK Medical and Health Sciences University, Ras Al Khaimah P.O. Box 11172, United Arab Emirates; vijaypaul@rakmhsu.ac.ae; 4 Department of Biochemistry, RAK College of Medical Sciences, RAK Medical and Health Sciences University, Ras Al Khaimah P.O. Box 11172, United Arab Emirates; grisilda@rakmhsu.ac.ae; 5 Department of Pharmacology, RAK College of Medical Sciences, RAK Medical and Health Sciences University, Ras Al Khaimah P.O. Box 11172, United Arab Emirates

**Keywords:** *Lacticaseibacillus rhamnosus*, *Lactobacillus rhamnosus*, colorectal cancer, in vitro, cell culture, cellular model, cancer cell line

## Abstract

This systematic review aimed to synthesize experimental evidence on the anticancer effects of *Lacticaseibacillus rhamnosus* (*L. rhamnosus*) and its derivatives against colorectal cancer (CRC) cell models. Eligible studies investigated probiotics, postbiotics, or bioactive compounds derived from *L. rhamnosus* with an in vitro component; studies relying solely on in vivo animal models, clinical trials, or observational designs were excluded. PubMed and Scopus were searched to identify relevant studies. Risk of bias was assessed using a modified QUIN tool, and extracted data were tabulated. Owing to incomplete numerical data, meta-analysis was not feasible, and the results were synthesized accordingly. Seventeen studies were included. *L. rhamnosus* and its derivatives reduced CRC cell proliferation, induced apoptosis, and caused cell cycle arrest. Reported mechanisms included upregulation of Bax, caspase-3/9, and p53; downregulation of Bcl-2/Bcl-xl; inhibition of Wnt/β-catenin signaling; reduced invasion and migration; increased reactive oxygen species; and immunomodulatory effects. Key limitations were heterogeneity in interventions, dosages, exposure periods, and cell lines, along with incomplete reporting, which precluded quantitative synthesis. Overall, preclinical evidence indicates multimodal anticancer effects of *L. rhamnosus* in CRC models; however, standardized reporting and translational research are required.

## 1. Introduction

Colorectal cancer (CRC) remains a major global health burden, being the second leading cause of cancer-related deaths in the United States, where more than 1700 Americans died from cancer-related causes every day in 2019. While incidence and mortality rates for CRC have stabilized in the United States, global trends indicate that this burden is increasing, with incidence projected to rise by nearly 60% by 2030. The recent advent of immunotherapy has revolutionized cancer treatment, but its clinical success in treating CRC remains limited. Only about 10% of CRC patients are currently eligible for immunotherapy, and more than 50% exhibit resistance to treatment, largely due to impaired antitumor immune responses. In fact, nearly 95% of CRC patients are resistant to immunotherapy, highlighting the urgent need to develop adjuvant strategies that enhance antitumor immunity and reduce tumor size [1].

Gut microbiota plays a crucial role in human health. Gut bacteria facilitate the digestion and absorption of nutrients while simultaneously preventing the colonization of the intestinal epithelium by pathogens. Therefore, probiotics, which are live microorganisms beneficial to human health when consumed in sufficient quantities, gained significant attention for their anti-infective and anti-carcinogenic properties [2].

Given the close interaction between gut microbiomes and host immune regulation, there is growing interest in exploring dietary probiotic-based interventions as combination therapies to improve the effectiveness of immunotherapy in colorectal cancer [3].

Among these bacteria, *Lactobacillus rhamnosus*, recently reclassified as *Lacticaseibacillus rhamnosus*, has emerged as a promising candidate due to its potential to modulate immunity and fight cancer. *L. rhamnosus* has been shown to enhance the antitumor efficacy of 5-fluorouracil in CRC by significantly reducing K-ras and Treg/IL-10 transcript levels, along with improving both innate and adaptive immune responses [4]. Similarly, the M9 strain of *L. rhamnosus* has shown its ability to enhance anti-tumor responses to anti-PD-1 therapy by modifying intestinal metabolites [5]. In preclinical models, the AFY06 strain has been reported to alleviate weight loss, increase organ indices, reduce the incidence of bowel tumors, improve pathological tissue damage, and suppress inflammatory signaling by downregulating molecules associated with NF-κB (IκBβ, p65, p50, and p52) and anti-apoptotic genes (Bcl-2 and Bcl-xL) and increasing pro-apoptotic mediators such as Bid and caspase-8 [6]. Furthermore, exopolysaccharides produced by several strains of Lactobacilli have been shown to inhibit the growth of HT-29 CRC cells by inducing G0/G1 phase cell cycle arrest and apoptosis [7]. This systematic synthesis examines original laboratory studies (in vitro) up to 30th June 2025 that evaluate *L. rhamnosus* and its derived products against CRC cell models to summarize mechanistic themes and identify evidence gaps to guide future translational research.

## 2. Materials and Methods

### 2.1. Eligibility Criteria

#### 2.1.1. Inclusion Criteria

1. Investigate probiotics, postbiotics, or bioactive compounds derived from *Lacticaseibacillus rhamnosus*/*Lactobacillus rhamnosus*.
2. Address colorectal cancer as the target disease model.
3. Report at least one relevant anticancer outcome, such as cell proliferation, apoptosis, cell cycle arrest, or tumor growth inhibition.
4. Original peer-reviewed research articles using laboratory-based (in vitro) CRC cell models.
5. Published from inception up to 30 June 2025.
6. Articles published in English. Articles in other languages may be included if a reliable English translation is available.
7. Studies combining *Lacticaseibacillus rhamnosus*/*Lactobacillus rhamnosus* with prebiotics are included, provided that the probiotic effect is a core focus.
8. Studies that contain both in vitro and in vivo components are included only if the in vitro data are clearly separated and independently extractable.
9. Studies involving combinations of different Lactobacillus species with *Lacticaseibacillus rhamnosus* will be included only if the outcomes for them are reported separately and can be independently extracted.

#### 2.1.2. Exclusion Criteria

1. Studies will be excluded if *Lacticaseibacillus rhamnosus*/*Lactobacillus rhamnosus* is not used as part of the intervention.
2. Rely solely on in vivo animal models, human clinical trials, or observational studies without an in vitro component.
3. Not included if studies do not involve probiotics, postbiotics, or bioactive microbial compounds derived from *Lacticaseibacillus rhamnosus*/*Lactobacillus rhamnosus.*
4. Investigate cancers other than colorectal cancer.
5. If it includes non-peer-reviewed sources, such as conference abstracts, editorials, dissertations, or grey literature.
6. Published in languages other than English, with no available English translation.
7. Assess *Lacticaseibacillus rhamnosus*/*Lactobacillus rhamnosus* in combination with non-dietary or pharmacological therapies (e.g., chemotherapeutics, radiation, herbal extracts), unless the effect of *Lacticaseibacillus rhamnosus*/*Lactobacillus rhamnosus* is evaluated independently and reported separately.
8. Lack relevant control groups or appropriate experimental design controls.
9. Do not report cell proliferation, apoptosis, tumor suppression, or other anticancer mechanisms as an outcome of their study.
10. Present in vitro and in vivo results in a combined or inseparable manner, preventing accurate extraction of in vitro findings.
11. Studies published after 30 June 2025.

### 2.2. Study Selection Process

All included studies were screened against the inclusion/exclusion rules and accepted as the included set because they met the specified criteria and were included in the final dataset [8,9,10,11,12,13,14,15,16,17,18,19,20,21,22,23,24]. Studies were selected from PubMed and Scopus using the following search terms: (*Lactobacillus rhamnosus* OR probiotics OR *Lacticaseibacillus rhamnosus*) AND “colorectal cancer” AND (“in vitro” OR “cell culture” OR “cellular model “ OR “cancer cell line”).

A.N.B.S., M.M.A., and A.S. independently screened studies retrieved from PubMed, while S.M.V., S.N.F., L.K., and E.H.N.F. independently screened studies obtained from Scopus. Screening was conducted manually in the following stages: deduplication, title screening, abstract screening, and full-text screening. The number of studies at each stage was recorded in accordance with PRISMA guidelines. Any disagreements were resolved through discussion, and unresolved conflicts were adjudicated by S.K.S.

### 2.3. Data Extraction and Synthesis of Evidence

The extracted elements included authors/year, cell line(s), methodology, outcomes (proliferation, apoptosis, cell cycle, invasion/migration, and molecular markers), and results [8,9,10,11,12,13,14,15,16,17,18,19,20,21,22,23,24]. Due to inconsistent numerical reporting across studies, the findings were summarized qualitatively, with quantitative values included only when explicitly provided. Data was manually extracted from full texts into a structured spreadsheet. Entries were cross-checked by multiple reviewers. The table is intentionally narrative and non-analytic, without subgrouping or statistical synthesis (Table 1). Due to substantial heterogeneity, a quantitative meta-analysis was not feasible. Therefore, a vote-counting approach based on the direction of effect was applied, whereby each study was categorized according to whether it reported evidence supporting specific anticancer mechanisms (e.g., proliferation inhibition, apoptosis induction, cell-cycle arrest, etc.). The number of studies demonstrating each mechanism was then summarized as *n*/N across the included studies (Table 2).

### 2.4. Quality Assessment (Risk of Bias)

A methodological quality assessment of the 17 included in vitro studies was conducted using a custom 11-item checklist adapted from the QUIN (Quality Index for In Vitro Studies) guideline [25]. Although the original QUIN tool was developed during in vitro dental research, it was modified to improve relevance to cancer cell line-based experimental models. However, there are no validated or consensus risk-of-bias tools currently existing for in vitro cell culture studies; hence, adapted frameworks are commonly used in laboratory systematic reviews. The adapted tool assessed key domains related to study aims, cell line characteristics, experimental design, outcome measurement, statistical analysis, and reporting transparency. Each criterion was scored on a three-point scale (0 = not met, 1 = partially met, and 2 = fully met), yielding a maximum possible score of 22. Study-level quality scores are presented in a dedicated table (see Supplementary Materials for quality analysis heat map). The mean quality score across included studies was 19.5 ± 1.79, indicating generally high methodological and reporting quality. While all studies clearly stated their objectives and reported outcomes transparently, variability was observed in reporting cell line authentication, statistical details, and the discussion of limitations. Quality scores were used to aid the interpretation of findings rather than as exclusion criteria.

### 2.5. Study Registration and Reporting

**Figure 1:**
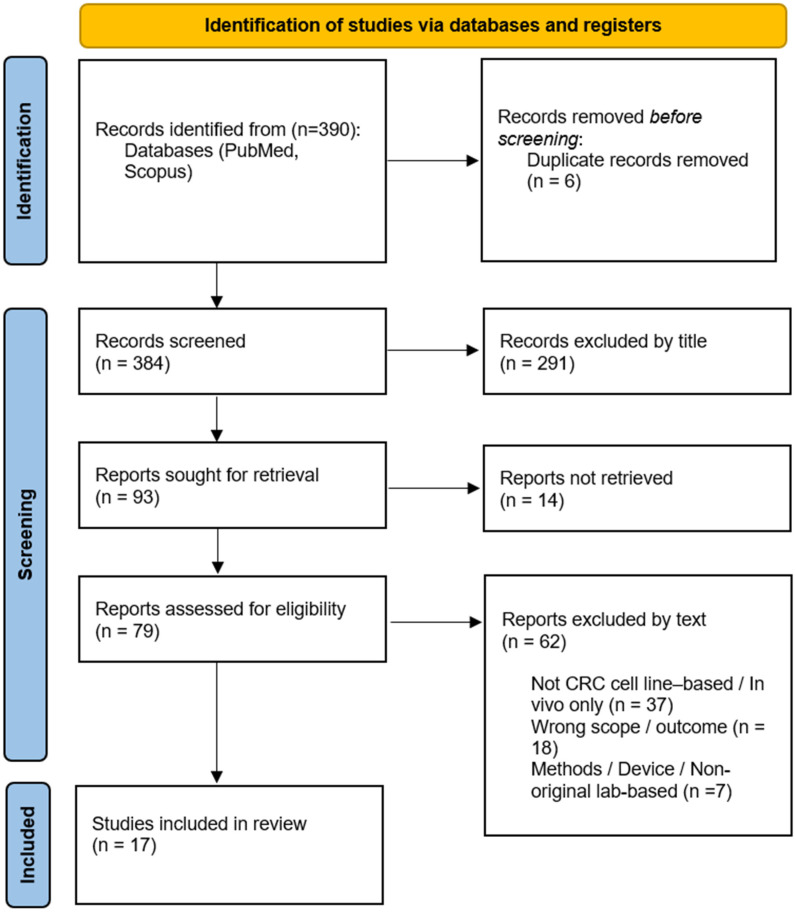
The systematic review (OSF; registration ID: 3jgae) was conducted according to the latest PRISMA guidelines. Records were identified from two databases, with six duplicate records removed before screening. A total of 384 records were screened based on their titles, and 291 were excluded. Of the 93 requested reports, 14 were not obtained. Seventy-nine full-text articles were assessed for eligibility, and 62 were excluded due to being combination studies, limited to studies conducted on living organisms, or using non-original laboratory methods. A total of 17 studies met the inclusion criteria and were analyzed.

This systematic review was registered retrospectively with the Open Science Framework (OSF) Registry, available from: https://osf.io/3jgae/overview (accessed on 11 March 2026). This study had already progressed substantially before the authors became aware of the registry requirement. No changes were made to the research question, eligibility criteria, outcomes, or analysis methods. Registration is reported here for transparency. This study abided by the reporting guidelines of PRISMA (Figure 1).

## 3. Results

### 3.1. Literature Search and Study Selection

The supplied set included 17 studies spanning 2012–2025, covering common CRC cell lines (HT-29, HCT-116, Caco-2, and DLD-1) with interventions including live *L. rhamnosus* strains, heat-killed cells, cell-free supernatants (CFSs), postbiotics, exopolysaccharides (EPSs), isolated proteins (p8), and engineered particles (Table 1).

### 3.2. Summary of Experimental Design and Methods of Studies Included

- Types of *L. rhamnosus* Interventions Studied: Live *L. rhamnosus* strains and variants [13,20,22,23], heat-killed/paraprobiotics [11,22], cell-free supernatants, postbiotics [12,13,18,22], isolated proteins (p8 and r-p8) [8,14,16], EPS [24], and nanoparticles/microparticles [17].
- Cellular and Experimental Models Employed: HT-29, HCT-116, Caco-2, DLD-1, HGC-27 (gastric in one study), primary CRC spheroids, and several normal control lines (HIEC-6, HEK-293, and HaCaT) used for selectivity testing [11,13,19,20,21,24].
- Assays and Outcome Measures Used to Evaluate Anticancer Effects: MTT/CCK-8/AlamarBlue (viability/proliferation) [12,17,19,20,22,23,24]; Annexin V/PI, AO/EB staining (apoptosis) [8,12,16,17,19,24]; flow cytometry cell-cycle analysis; caspase activity assays and Western blot/qRT-PCR for pathway proteins [8,12,19,24]; invasion/migration and MMP-9 zymography [15].
- Under the predefined criteria, full-text articles were assessed for eligibility. Studies were excluded if they did not specifically evaluate *L. rhamnosus* in an in vitro CRC model with relevant anticancer endpoints. Excluded studies involved those describing 3D drug-testing platforms [26], biomaterial-based drug delivery systems [27], a device development smart capsule study [28], and organ-on-chip models without probiotic intervention or CRC cell-line anticancer assessment [29]. In total, 62 full-text articles were excluded for not meeting the specified experimental design requirements.

### 3.3. Anticancer Mechanism of L. rhamnosus

#### 3.3.1. Inhibition of Cancer Cell Growth

Numerous studies consistently reported that *L. rhamnosus* and its preparations reduce the proliferation of CRC cells in vitro. *L. rhamnosus* GG (LGG) CFS inhibited proliferation in HT-29 and Caco-2 cells, demonstrating both dose- and time-dependent effects [12]. Culture of live LGG cells resulted in a significant reduction in the growth of HT-29 and HCT-116 cells, confirming the potential for replicating antiproliferative effects across cell lines [20]. Similarly, ethyl acetate extracts of *L. rhamnosus* ATCC 7469 decreased Caco-2 cell proliferation, while EPS of *L. rhamnosus* effectively inhibits HT-29 and HCT-116 colony formation in a dose-dependent manner [19,24]. Engineered formulations, such as Ag-LNPs manufactured using *L. rhamnosus* lysate, have also shown strong antiproliferative effects, sometimes exceeding the activity of raw live cultures [17]. These findings highlight that both live and derivative *L. rhamnosus* products can consistently reduce CRC cell growth.

*L. rhamnosus* and its derivatives induce apoptosis primarily via the intrinsic mitochondrial pathway. Treatment with the r-p8 protein increased Bax/Bcl-2 ratios and caspase-3/9 cleavage in DLD-1 and HCT-116 cells, leading to DNA fragmentation and apoptosis [8]. LGG CFS similarly activated caspases and induced apoptosis in HCT-116 cells [15]. Postbiotics derived from *L. rhamnosus* bacteria increased Bax and caspase-3 expression in HT-29 cells, reinforcing the idea that soluble bioactive components can induce programmed cell death [18]. Taken together, these studies indicate that induction of programmed cell death is a robust and reproducible mechanism of *L. rhamnosus* anticancer activity, and this has been observed across both protein-derived and polysaccharide-derived biologically active fractions.

#### 3.3.2. Suppression of Metastatic Potential

*L. rhamnosus* and its derivatives also subdue metastatic phenotypes in CRC cell models. LGG CFS resulted in decreased HCT-116 cell migration and MMP-9 activity, indicating reduced invasive capacity [15]. r-p8 treatment reduced migration in DLD-1 cells, correlating with cytoskeletal remodeling [16].

*L. rhamnosus* interventions affect multiple major oncogenic gene pathways. A persistent decrease in Wnt/β-catenin signaling has been observed in drug-resistant strains, resulting in reduced levels of β-catenin, c-Myc, and Cyclin D1 in HT-29 and HCT-116 cells [14]. The recombinant p8 protein has been shown to upregulate RNF152, a mediator of apoptosis, highlighting the involvement of additional protein-specific mechanisms [8]. Taken together, these data show that the biologically active substances in *L. rhamnosus* can interfere with cell movement and the degradation of the extracellular matrix, suggesting an anti-metastatic potential.

#### 3.3.3. Disruption of Cellular Metabolism and Microenvironment

*L. rhamnosus* interventions also target mitochondrial function and oxidative stress. LGG treatment reduced mitochondrial respiration and ATP production in HT-29 and HCT-116 cells, indicating energy disruption [20]. ROS accumulation observed with EPS treatment further promoted apoptotic signaling [24]. These results suggest that *L. rhamnosus* components can impair energy metabolism and induce oxidative stress to promote cancer cell death.

*L. rhamnosus* derivatives modulate host immune responses, potentially enhancing antitumor immunity. Probiotic extract treatments increased IFN-γ, TNF-α, and IL-17A levels in HT-29 cells while preserving normal cells [21]. Probiotic and postbiotic treatments also led to a reduction in pro-inflammatory cytokines and an increase in interleukin-10 in Caco-2 models [22,23]. These results suggest that stem-cell-related interventions may modify the tumor microenvironment and host immunity.

#### 3.3.4. Selective Cytotoxicity and Translational Potential

Several studies have demonstrated the selective toxicity of *L. rhamnosus* preparations against cancer cells while preserving normal cells. Live and cell-free supernatant *L. rhamnosus* strains reduced Caco-2 cell proliferation but had little effect on HIEC-6 cells [13]. Similarly, ethyl acetate extracts and postbiotics showed low cytotoxicity in HUVEC, HEK-293, and HaCaT cells [18,19]. Collectively, these results elicit a favorable safety profile and selective anticancer activity.

### 3.4. Comparative Effectiveness of the Probiotic Formulations

Across the included studies, the formulations that demonstrated the strongest and most consistent anticancer effects were CFS and EPS. CFS from *L. rhamnosus* repeatedly showed clear reductions in CRC cell viability, suppression of growth, and enhanced anticancer activity across multiple cell lines [12,15,18,22,23]. EPS preparations likewise produced a reliable decrease in proliferation and strong inhibitory effects on HT-29 and HCT-116 cells across different experiments, making them one of the most reproducible preparations in the dataset [24]. Among the remaining groups, P8-based derivatives (P8, r-P8, and PP-P8) showed substantial anticancer effects but were evaluated in fewer studies, placing them slightly below CFS and EPS in overall consistency [8,14,16]. Live *L. rhamnosus* also demonstrated meaningful anticancer activity, though results varied more by strain and study conditions [13,20]. Nanoparticle- and microparticle-based *L. rhamnosus* derivatives showed promising results but were limited to individual studies [17]. Overall, CFS and EPS stand out as formulations with the most consistent and robust anticancer effects across the included evidence base.

### 3.5. Vote Counting Synthesis of Anticancer Mechanisms of L. rhamnosus

This systematic review employed a vote-counting approach to synthesize evidence on the anticancer mechanisms of *L. rhamnosus*. Studies were categorized based on reduced cancer cell proliferation/viability, apoptosis induction, cell cycle arrest, inhibition of migration/invasion, modulation of oncogenic signaling pathways (Wnt/β-catenin, p53, p21, Cyclin B1/Cdk1), immune/inflammatory modulation, mitochondrial dysfunction/ROS-mediated apoptosis, and selective cytotoxicity. The frequency of studies supporting each mechanism was tabulated and compared. This approach provided a structured qualitative summary in the absence of sufficient homogeneity for meta-analysis.

### 3.6. Summary of the Results

The result of our research demonstrates that *L. rhamnosus* strains and their non-viable derivatives (postbiotics, paraprobiotics, and CFS) exert multifaceted anticancer effects against CRC. These effects are primarily mediated by inducing apoptosis and cell cycle arrest through modulation of key pathways such as the Wnt/β-catenin pathway, the p53 pathway, and caspase activation [8,14,24]. These interventions also consistently inhibit the proliferation, migration, and invasion of cancer cells, while exhibiting selective toxicity towards malignant cells compared to normal cells [9,13,15,20,21]. Furthermore, it promotes a beneficial immune shift, characterized by a decrease in pro-inflammatory cytokines (such as IL-6 and TNF-α) and an increase in anti-inflammatory mediators such as IL-10 [10,13,22,23].

## 4. Discussion

This review synthesizes in vitro evidence demonstrating that *L. rhamnosus* and its derivatives exert consistent, multi-mechanistic anticancer effects against CRC cell lines. Across 17 studies, live cells, CFS, EPS, purified proteins (e.g., p8), and engineered formulations (nano/microparticles) were shown to inhibit proliferation, induce apoptosis and cell-cycle arrest, disrupt mitochondrial function, suppress invasion and migration, modulate Wnt/β-catenin and TGF-β/BMP pathways, and promote an anti-inflammatory milieu. A notable and recurring finding is selective cytotoxicity against malignant cells with minimal effects on normal epithelial or fibroblast cells.

Specific studies further elucidate these mechanisms. Salemi et al. (2023) demonstrated that LGG CFS selectively reduced cancer cell viability via G2/M arrest and enhanced sensitivity to 5-fluorouracil and irinotecan [30]. Zhao et al. (2023) showed that *L. rhamnosus* strains strengthened intestinal barrier integrity through modulation of claudin-3, occludin, and E-cadherin, while LGG also inhibited proliferation and induced apoptosis through Bax, caspase-3, and p53 upregulation and reductions in inflammatory proteins [31]. Gai et al. (2023) reported successful colonization of the intestine by *L. rhamnosus* in healthy adults with the suppression of harmful bacterial pathways, supporting the role of microbial stabilization relevant to protection against CRC [32].

Although not central to our aim, adjunctive clinical evidence supports the wider relevance of probiotics in CRC care. Huang et al. (2023) observed reduced chemotherapy-induced diarrhea and restoration of microbial diversity, while Thomsen et al. (2024) reported improved bowel function, microbial stability, and quality of life with multi-strain probiotics during cancer therapy [33,34]. These results demonstrate that probiotics have both direct anticancer effects and a supporting role in preserving gut homeostasis.

Methodologically, this review differs from prior analyses (Zhong et al., Sanchez et al., Gao et al.) by focusing exclusively on *L. rhamnosus*, integrating emerging postbiotic evidence and applying a structured quality assessment adapted from QUIN guidelines [35,36,37]. This approach provided guidance for future research quality improvement by identifying recurring reporting gaps, especially in cell lineage documentation and limitation reporting.

In comparison with earlier systematic reviews that broadly addressed probiotics and CRC mechanisms (Zhong et al., Gao et al.), our findings confirm established pathways such as apoptosis induction and Wnt/β-catenin modulation while adding depth through identification of *L. rhamnosus*-specific effectors [35,37]. Notably, the role of p8 in G2/M arrest via the p53–p21 axis and RNF152 upregulation, the consistent modulation of cytokine profiles (↓ IL-1, TNF-α, IFN-γ; ↑ IL-10, IL-12), the inhibition of metastasis-related markers (MMP-9, ANXA9), and the exploration of advanced delivery systems represent key additions to existing knowledge.

Our study also highlights specific areas where reporting practices could be improved to enhance reproducibility and transparency. This includes providing complete documentation of cell lineages, which were fully achieved in half of the studies. Gaps were also noted in the incomplete identification of treatment doses and exposure durations, as well as in the comprehensive description of statistical methods. An opportunity to enhance scientific rigor lies in the more consistent inclusion of a section dedicated to limitations, as this crucial aspect of study interpretation was not fully addressed in many reports.

Collectively, the data point to a logical model in which *L. rhamnosus* and its derivatives stabilize gut barrier and microbiota functions while exhibiting selective, multi-targeted anticancer activity, thereby enhancing their potential as precise, biologically active agents in the prevention and treatment of colorectal cancer.

### 4.1. Limitations

It must be acknowledged that this review has several limitations, with many stemming from inherent constraints in the primary studies included [8,9,10,11,12,13,14,15,16,17,18,19,20,21,22,23,24]. The most significant limitation is the wide variability among the formulations, which ranged from live bacteria and crude supernatants to highly purified proteins and synthetic nanomaterials. This variability, along with differences in dosage, exposure periods, and cell line models, prevented a meaningful quantitative meta-analysis, a challenge also observed in other probiotic reviews [36]. Since this is a systematic review of in vitro studies, significant methodological and outcome heterogeneity precluded quantitative synthesis, subgroup analysis, sensitivity analysis, and formal heterogeneity or reporting bias assessments. The review protocol was registered retrospectively on OSF. While prospective registration is encouraged for clinical systematic reviews, formal registry requirements for in vitro systematic reviews remain less standardized. Additionally, due to the absence of consensus guidelines specifically for risk of bias and certainty assessment in in vitro studies, comprehensive bias modeling and evidence grading could not be fully performed. Our quality assessment revealed specific weaknesses in the published studies: Only 50% provided detailed documentation of the cell lines, while a third offered an incomplete description of the statistical methods or dose/exposure parameters. Crucially, a discussion of study limitations was absent or inadequate in approximately one-third of the reports, hindering a critical assessment of potential biases. These limitations included the selection of specific cell lines that responded, the lack of physiological relevance in monolayer cell culture systems, and the general absence of experiments controlling for the effects of bacterial medium components or pH changes in CFS treatments. These reporting gaps and methodological inconsistencies limit the reproducibility, comparability, and practical applications of the findings. Therefore, our findings should be interpreted considering these limitations.

### 4.2. Prospects

This analysis identifies clear pathways for advancing this field. Future research should prioritize several key areas. First, standardization is needed, particularly in establishing agreed-upon definitions and purification criteria for cell-derived biomaterials and extracellular polymers, to enable direct and meaningful comparisons between studies. Second, the field must move toward improved model systems that go beyond traditional two-dimensional single-cell cultures. The use of more physiologically appropriate models, such as three-dimensional organelles, patient-derived globules, and microfluidic gut-on-a-chip co-cultures incorporating immune cells and microbiome components, will provide more accurate predictive data for human biology. Third, a deeper understanding of the mechanisms is essential. This can be achieved by employing multimodal approaches (proteomics and metabolomics) within these advanced models to identify the precise molecular sequences initiated by specific *L. rhamnosus* derivatives, moving from correlational observations to causal understanding. Furthermore, future research should aim to develop standardized and validated quality assessment tools specifically for in vitro cell culture studies. Addressing these priorities will be crucial for translating the promising multimodal anticancer activity of *L. rhamnosus* bioactive compounds, as detailed in this review, into reliable complementary strategies for the prevention and treatment of colorectal cancer.

## 5. Conclusions

Preclinical evidence from 17 studies suggests that *L. rhamnosus* and its bioactive derivatives may exert multiple anticancer-related effects in colorectal cancer (CRC) experimental models [8,9,10,11,12,13,14,15,16,17,18,19,20,21,22,23,24]. These studies report reductions in cell proliferation, induction of apoptosis and cell-cycle arrest, inhibition of migration and invasion, and modulation of cancer- and immune-related pathways. However, these findings are derived primarily from in vitro systems with heterogeneous experimental conditions and therefore require further validation in standardized preclinical and clinical settings.

## Figures and Tables

**Table 1:** Summary of included in vitro studies evaluating anticancer potential of *L. rhamnosus*.

Author & Year	Cell Line	Methodology	Outcome	Results
Kim et al. (2021) [8]	DLD-1, HCT 116	Following RNF152 silencing, CRC cells were treated with recombinant p8. Apoptosis (flow cytometry, Western blot), gene expression (RT-PCR), and dose-dependent viability were measured.	r-p8 triggers apoptosis in CRC cell lines by increasing RNF152, which promotes apoptotic signaling pathways.	Following RNF152 silencing, r-p8 induced apoptosis (cleaved PARP1/caspase-3, Annexin V/PI) was significantly reduced, confirming RNF152 as essential for its pro-apoptotic activity.
Avcı et al. (2025) [9]	HT-29, Caco-2	Cytotoxicity was assessed using MTT and miRNA expression.	Probiotic-derived postbiotics selectively inhibit CRC cell growth and modulate miRNA expression.	Postbiotics of *L. rhamnosus* showed selective cytotoxicity against HT-29/Caco-2 cells with minimal effects on normal fibroblasts.
Erfanian et al. (2025) [10]	HT-29	Anti-inflammatory effects of CFSs extracted from cultured *L. Rhamnosus* were investigated by qRT-PCR.	CFS from *L. rhamnosus* exhibited anti-inflammatory properties in vitro (decreased IL-6 & TNF-α).	Microbial imbalances may serve as early CRC biomarkers, with probiotic-derived CFSs offering a non-invasive strategy for managing CRC-associated inflammation.
Orlando et al. (2012) [11]	HGC-27, DLD-1	DLD-1 CRC cells were treated with viable cells, and heat-killed CRC cells were treated with *Lactobacillus rhamnosus* GG; proliferation (MTT) and apoptosis (ELISA, Annexin V/flow cytometry) were assessed.	Antiproliferative and pro-apoptotic effects on CRC cells.	Both viable and heat-killed *Lactobacillus rhamnosus* GG reduced DLD-1 proliferation and increased apoptosis within 24–48 h.
Si et al., 2022 [12]	HT-29, Caco-2	CFS from LGG was prepared and assessed for cytotoxicity (MTT), apoptosis (Annexin V/PI flow cytometry), and apoptotic markers (via Western blot).	Outcomes included reduced cell viability, induced apoptosis and cell cycle arrest, and modulated apoptotic protein expression.	Treatment reduced viability in HT-29/Caco-2 cells, induced apoptosis (Annexin V+), triggered G0/G1 arrest, and modulated apoptotic markers (↑ caspase-3, ↑ Bax, ↓ Bcl-2).
Pahumunto et al., 2023 [13]	Caco-2, HIEC-6	*L. rhamnosus* SD4/SD11/GG and their CFS were tested for anti-bacterial, anti-inflammatory, and anti-cancer activity, including defensin/IL-10 expression and intestinal survival.	SD11 selectively inhibited cancer cells, reduced inflammation, and enhanced IL-10/β-defensin expression, while all strains showed good intestinal survival and adhesion, supporting their probiotic potential.	SD11 exhibited selective anti-cancer and anti-inflammatory activity, while SD4 showed the strongest intestinal adhesion and all strains survived gut conditions, highlighting strain-specific functional benefits.
An et al., 2023. [14]	DLD-1	P8 protein from *L. rhamnosus* was delivered via *Pediococcus pentosaceus* (PP-P8) and assessed in vitro for proliferation, cell cycle, and target identification (pull-down, LC-MS/MS, NGS).	PP-P8 inhibited proliferation, induced G2 arrest, and disrupted Wnt signaling via GSK3β interaction and β-catenin phosphorylation.	P8 induces G2 arrest via CDK1/Cyclin B1 downregulation and disrupts Wnt signaling by binding GSK3β to promote β-catenin degradation and alter its transcription.
Escamilla et al., 2012 [15]	HCT-116	HCT-116 cells were treated with LGG CFS and assessed for invasion, MMP-9 expression/activity, and ZO-1 levels. CFS fractionation was performed to identify active compound sizes.	LGG CFS reduced cancer cell invasion and MMP-9 activity while increasing ZO-1 expression. Anti-invasive effects localized to high molecular weight fractions (>50 kDa), suggesting macromolecular effectors.	LGG CFS inhibited CRC cell invasion, reduced MMP-9, and enhanced ZO-1, indicating anti-metastatic potential via high-molecular-weight bioactives.
An et al., 2019 [16]	DLD-1	Cells were treated with r-p8 or transfected with p8 plasmid, and protein expression (Western blot), localization (confocal microscopy), and cell cycle (flow cytometry) were analyzed.	There is reduced cell growth and migration with G2 arrest, associated with blockade of the p53–p21–Cyclin B1/Cdk1 pathway.	Recombinant P8 reduces cell growth by 20% and migration by 44%, while endogenous P8 is roughly twice as potent, inducing G2 arrest and blocking the p53–p21–Cyclin B1/Cdk1 pathway.
Aziz Mousavi et al. 2020 [17]	HT-29	Silver nanoparticles were synthesized using *L. rhamnosus* GG lysate, characterized (TEM, FTIR, XRD), and assessed for anticancer activity in HT-29 cells via MTT and Annexin/PI staining.	Ag-LNPs reduced HT-29 viability and induced apoptosis via ROS generation and mitochondrial damage.	LGG-synthesized AgNPs induced dose-dependent apoptosis in HT-29 cells via ROS and mitochondrial pathways, demonstrating stable anticancer potential.
Erfanian et al., 2024 [18]	HT-29	*L. rhamnosus* postbiotics were assessed for anti-proliferative and anti-migratory effects in HT-29 cells (MTT, scratch assay) and for apoptosis via Bax, Bcl-2, and caspase-3 expression.	*L. rhamnosus* postbiotics demonstrate moderate anticancer potential by reducing proliferation and migration and modulating apoptosis-related markers.	*L. rhamnosus* postbiotics induced anti-proliferative, anti-migratory, and pro-apoptotic effects in HT-29 cells.
Amin et al., 2023 [19]	Caco-2	The ethyl acetate extract of *L. rhamnosus* ATCC 7469 was tested using an MTT assay for viability, Annexin V/PI flow cytometry for apoptosis, caspase-3/8/9 activity assays, and qRT-PCR for apoptosis-related genes (bcl-2, bcl-xl, bax, bak, bad).	There is an increase in caspase-3/8/9 and Bax, along with reduced Bcl-2, indicating apoptosis through both intrinsic and extrinsic pathways and decreased Caco-2 viability.	*L. rhamnosus* extract reduces Caco-2 viability by 71% at 48 h, increases caspase-3/9 and bax/bak/bad, and decreases bcl-2/bcl-xl, indicating a time- and dose-dependent, tumor-selective intrinsic apoptotic response.
Budu et al., 2023 [20]	HaCaT, HT-29 and HCT-116	LGG was cultured in MRS broth/agar, and its effects were assessed using Alamar Blue for viability, DAPI staining for nuclear changes, colorimetric assays for BAX, Bcl-2, caspase-3, and Cyclin D1, and high-resolution respirometry to evaluate mitochondrial function.	*L. rhamnosus* GG reduces CRC cell growth, increases apoptosis (↑ Bax, ↑ caspase-3, ↓ Bcl-2, ↓ Cyclin D1), and impairs mitochondrial function, while selectively sparing normal HaCaT cells.	*L. rhamnosus* GG decreases HT-29 and HCT-116 viability within 24–48 h with minimal impact on HaCaT cells, accompanied by increased Bax and caspase-3, reduced Bcl-2, nuclear condensation, and mitochondrial dysfunction (reduced respiration, OXPHOS, and ATP).
Salek, S., et al. (2024) [21]	HT-29,	LGG was tested using an MTT assay for cell viability, AO/EB staining for apoptosis, and flow cytometry to assess Th17 cytokine production.	HT-29 cells showed decreased viability, increased apoptosis, and elevated Th17 cytokines (IFN-γ, TNF-α, IL-17A), while HEK-293 cells demonstrated increased viability and reduced apoptosis.	↑ IFN-γ, TNF-α, and IL-17A indicate an enhanced anti-tumor and pro-inflammatory immune response.
Avci, G.A., et al. (2024) [22]	Caco-2	Live *L. rhamnosus* preparations: probiotics, Para probioticss, and postbiotic CFS. Evaluated using MTT for viability and ELISA for IL-1, IL-10, IL-12, IL-13, TNF-α, IFN-γ, and neopterin.	It showed dose-dependent cytotoxicity in cancer cells. There was a shift toward reduced pro-inflammatory and increased anti-inflammatory cytokines.	All preparations reduced Caco-2 viability; postbiotics strongest. Shows decreased IL-1, TNF-α, IFN-γ, and neopterin while increasing IL-10, IL-12, and IL-13.
Viana, R., et al. (2024) [23]	Caco-2	Live *L. rhamnosus* cultures in MRS were tested using MTT for viability and ELISA for IL-1, IL-10, IL-12, IL-13, TNF-α, IFN-γ, and neopterin.	Primary: cytotoxicity of probiotics on Caco-2. Secondary: cytokine-mediated immune modulation, efficacy of live cultures.	Probiotics reduced Caco-2 viability and lowered IL-1, TNF-α, IFN-γ, and neopterin while increasing IL-10, IL-12, and IL-13.
Lee, Y.-J., et al. (2025) [24]	HT-29 HCT-116	*L. rhamnosus* EPS was tested using CCK-8 and colony formation assays, Annexin V/PI apoptosis analysis, cell-cycle and ROS assays, and qPCR/Western blotting for key markers.	Primary: reduced CRC proliferation and increased apoptosis and cell-cycle arrest. Secondary: elevated ROS and modulation of apoptosis and cell-cycle-related genes/proteins.	Upregulated: Bax, Caspase-3/9, p53, and ROS; downregulated: Bcl-2 and Cyclin D1.

**Table 2:** Vote counting summary of anticancer mechanisms of *L. rhamnosus* in CRC models. N = 17 represents the total number of included studies; percentages are presented as *n*/N, indicating the % of studies reporting each effect.

Anticancer Mechanism	Studies Supporting Effect (*n*)	Percentage (*n*/N)
Reduced cancer cell proliferation/viability	13	76.5%
Apoptosis induction	10	58.8%
Cell cycle arrest	4	23.5%
Inhibition of Migration/invasion	3	17.6%
Modulation of oncogenic signaling pathways (Wnt/β-catenin, p53, p21, Cyclin B1/Cdk1)	3	17.6%
Immune/inflammatory modulation	5	29.4%
Mitochondrial dysfunction/ROS-mediated apoptosis	3	17.6%
Selective cytotoxicity	4	23.5%

## Data Availability

No new data is generated by this review. All information is part of this manuscript.

## Supplementary Materials

The following supporting information can be downloaded at https://www.mdpi.com/article/10.3390/ijms27072944/s1.

## Abbreviations

The following abbreviations are used in this manuscript:

CFSs	Cell-free supernatants;
CRC	Colorectal cancer;
EPSs	Exopolysaccharides;
LGG	*L. rhamnosus* GG;
LNP	Lipid nanoparticle;
LR	*L. rhamnosus*.
