# Supplementary material for: Anticancer Potential of Lacticaseibacillus rhamnosus in Colorectal Cancer—A Systematic Review of In Vitro Cell Culture Evidence
Source: Int J Mol Sci. 2026 Mar 24;27(7):2944. doi: 10.3390/ijms27072944 (PMC13073571; doi:10.3390/ijms27072944)
Supplement: Supplementary file 1 [file ijms-27-02944-s001.zip › ijms-4169925-Supplementary.pdf]

Anticancer Potential of Lacticaseibacillus rhamnosus in Colorectal Cancer- A Systematic Review of In Vitro Cell Culture Evidence. S1. Quality analysis scoring for included studies

		Kim et al. (2021)	Avcı et al. (2025)	Erfanian et al. (2025)	Orlando et al. (2012)	Si et al. (2022)	Pahumunto et al. (2022)	An et al. (2023)	Escamilla et al. (2012)	An et al. (2019)	Aziz Mousavi et al. (2020)	Erfanian et al. (2024)	Amin et al. (2023)	Budu et al. (2023)	Salek et al. (2024)	Avci et al. (2024)	Viana et al. (2024)	Lee et al. (2025)
1	The study aims/objectives are clearly stated, with the research question or hypothesis explicitly defined.	2	2	2	2	2	2	2	2	2	2	2	2	2	2	2	2	2
2	Cell line/sample details are provided, including the name, source, authentication, and passage number.	2	2	2	2	1	1	1	2	2	1	2	2	2	1	1	2	1
3	Culture conditions are described, including medium composition, supplements, and incubation environment.	2	2	2	2	2	2	2	2	2	2	2	2	2	2	0	2	2

Green : completely described; Orange : partially described; Red : not mentioned

Anticancer Potential of Lacticaseibacillus rhamnosus in Colorectal Cancer- A Systematic Review of In Vitro Cell Culture Evidence. S1. Quality analysis scoring for included studies

		Kim et al. (2021)	Avcı et al. (2025)	Erfanian et al. (2025)	Orlando et al. (2012)	Si et al. (2022)	Pahumunto et al. (2022)	An et al. (2023)	Escamilla et al. (2012)	An et al. (2019)	Aziz Mousavi et al. (2020)	Erfanian et al. (2024)	Amin et al. (2023)	Budu et al. (2023)	Salek et al. (2024)	Avci et al. (2024)	Viana et al. (2024)	Lee et al. (2025)
4	Experimental methodology is clearly explained, with all methods described in sufficient detail for replication.	2	1	2	2	2	2	2	2	2	2	2	2	2	2	1	2	2
5	Control groups are specified, including the type(s) of controls used such as untreated, vehicle, or positive controls.	2	2	2	2	2	2	2	2	2	2	2	2	2	2	2	2	2
6	Dose and exposure time are clearly reported, with concentrations and duration of treatment specified.	2	1	2	2	2	1	2	1	2	2	2	2	2	2	0	2	2

Green : completely described; Orange : partially described; Red : not mentioned

Anticancer Potential of Lacticaseibacillus rhamnosus in Colorectal Cancer- A Systematic Review of In Vitro Cell Culture Evidence. S1. Quality analysis scoring for included studies

		Kim et al. (2021)	Avcı et al. (2025)	Erfanian et al. (2025)	Orlando et al. (2012)	Si et al. (2022)	Pahumunto et al. (2022)	An et al. (2023)	Escamilla et al. (2012)	An et al. (2019)	Aziz Mousavi et al. (2020)	Erfanian et al. (2024)	Amin et al. (2023)	Budu et al. (2023)	Salek et al. (2024)	Avci et al. (2024)	Viana et al. (2024)	Lee et al. (2025)
7	Outcome measurement methods are clearly described (e.g., MTT, PCR, Western blot).	2	2	2	2	2	2	2	2	2	2	2	2	2	2	1	2	2
8	Statistical analysis is appropriate, with tests, sample size, variability, and significance criteria reported.	1	1	2	2	2	2	2	2	1	2	2	2	2	2	2	2	2
9	Results are fully presented, with data clearly shown (mean ± SD/SEM, figures/tables) and all relevant findings reported.	2	1	2	2	2	2	2	2	2	2	2	2	2	1	2	2	2

Green : completely described; Orange : partially described; Red : not mentioned

Anticancer Potential of Lacticaseibacillus rhamnosus in Colorectal Cancer- A Systematic Review of In Vitro Cell Culture Evidence. S1. Quality analysis scoring for included studies

		Kim et al. (2021)	Avcı et al. (2025)	Erfanian et al. (2025)	Orlando et al. (2012)	Si et al. (2022)	Pahumunto et al. (2022)	An et al. (2023)	Escamilla et al. (2012)	An et al. (2019)	Aziz Mousavi et al. (2020)	Erfanian et al. (2024)	Amin et al. (2023)	Budu et al. (2023)	Salek et al. (2024)	Avci et al. (2024)	Viana et al. (2024)	Lee et al. (2025)
10	Limitations and potential biases are discussed, acknowledging study weaknesses, confounders, and in vitro limitations.	1	1	2	1	0	1	0	1	1	1	1	1	1	2	0	2	1
11	Conflict of interest and funding are disclosed, ensuring transparency about sources and potential conflicts.	2	2	1	1	2	2	2	2	2	2	2	2	2	1	2	2	2
	TOTAL SCORE	20	17	21	20	19	19	19	20	20	20	21	21	21	19	13	22	20

Green : completely described; Orange : partially described; Red : not mentioned
